# The effects of cognitive behavioral therapy on resting‐state functional brain network in drug‐naive patients with obsessive–compulsive disorder

**DOI:** 10.1002/brb3.963

**Published:** 2018-04-16

**Authors:** Ping Li, Xiangyun Yang, Andrew J. Greenshaw, Sufang Li, Jia Luo, Haiying Han, Jing Liu, Zhaoxi Zhong, Zhihua Guo, Hongfang Xiong, Shumin Yao, Yunhui Chen, Jing Sun, Zhanjiang Li

**Affiliations:** ^1^ Department of Psychiatry Qiqihar Medical University Qiqihar Heilongjiang China; ^2^ The China Clinical Research Center for Mental Disorders & Beijing Key Laboratory of Mental Disorders Beijing Anding Hospital Capital Medical University Beijing China; ^3^ Department of Psychiatry University of Alberta Edmonton AB Canada; ^4^ Department of Psychiatry & Behavioral Sciences Johns Hopkins University School of Medicine Baltimore MD USA; ^5^ Menzies Health Institute Queensland and School of Medicine Griffith University Nathan Qld Australia

**Keywords:** cognitive behavioral therapy, degree centrality, functional connectivity, obsessive–compulsive disorder, resting‐state fMRI

## Abstract

**Objectives:**

Although cognitive behavioral therapy (CBT) is an effective treatment for obsessive–compulsive disorder (OCD), the treatment mechanisms remain poorly understood. This study aimed to investigate the effects of CBT on changes in the intrinsic whole‐brain functional network of OCD patients.

**Materials and Methods:**

Twenty drug‐naive and noncomorbid OCD patients were recruited, and resting‐state functional magnetic resonance imaging was performed before and after 12 weeks of CBT. Moreover, 20 healthy controls were scanned twice with a 12‐week interval. A graph‐theory degree centrality (DC) approach and functional connectivity method were used to analyze the whole‐brain functional network hub and connectivity changes in OCD patients before and after CBT treatment.

**Results:**

A significant group × time interaction on DC was found in the left dorsolateral prefrontal cortex (DLPFC); the DC in the left DLPFC was significantly reduced after CBT treatment. Resting‐state functional connectivity (RSFC) between the left DLPFC and right orbitofrontal cortex was increased in the OCD patients at baseline, and normalized after CBT treatment. RSFC changes between the left DLPFC and default mode network (DMN) positively correlated with changes in clinical symptoms in OCD patients.

**Conclusions:**

These findings suggest that CBT can modulate changes in intrinsic functional network hubs in the cortico–striato–thalamo‐cortical circuit in OCD patients. Cognitive control network and DMN connectivity may be a potential imaging biomarker for evaluating CBT treatment for OCD.

## 1 INTRODUCTION

Obsessive–compulsive disorder (OCD) is a chronic disorder marked by recurrent, intrusive, distressing thoughts (obsessions) and/or repetitive behaviors (compulsions). OCD patients recognize the lack of practical relevance of their obsessions, yet are still compelled to engage in the rituals and repetitive behaviors, and are unable to dismiss their obsessional thoughts. Given the nature of the disorder, OCD is associated with abnormalities in or between default mode network, salience network, attention network and cognitive control network (Beucke et al., 2014; Greisberg & McKay, 2003; Menzies et al., 2008; Posner et al., 2017; Stern, Fitzgerald, Welsh, Abelson, & Taylor, 2012). Cognitive behavioral therapy (CBT) aims to restructure thought processes and regulate feelings and behavior, and can be an effective treatment for OCD (Rosa‐Alcazar, Sanchez‐Meca, Gomez‐Conesa, & Marin‐Martinez, 2008). Although CBT can result in a 40% to 60% improvement in the clinical symptoms of OCD, the underlying neural mechanism by which CBT exerts its effect remains unclear (Fisher & Adrian, 2005).

Several studies have provided evidence that CBT can affect the glucose metabolic rates, regional cerebral blood flow (rCBF) and blood oxygenation level dependence (BOLD) response of brain areas associated with the cortico–striato–thalamo‐cortical (CSTC) circuit in OCD patients, including the dorsolateral prefrontal cortex (DLPFC), orbitofrontal cortex (OFC), dorsal anterior cingulate cortex (dACC), caudate and thalamus (Morgieve et al., 2014; Nakao et al., 2005; Saxena et al., 2009; Schwartz, Stoessel, Baxter, Martin, & Phelps, 1996; Yamanishi et al., 2009; Yang et al., 2015). With a positron emission tomography approach, Saxena et al. (2009) discovered that OCD patients showed significant increased activity in the right dACC, yet significant decreased metabolism in the bilateral thalamus with intensive CBT (Saxena et al., 2009). Compared with poor responders of behavior therapy, Schwartz et al. (1996) found greater glucose metabolic rates of the bilateral caudate in OCD responders (Schwartz et al., 1996). Using single photon emission computed tomography, Yamanishi et al. (2009) found that the rCBF value in the left DLPFC was significantly lower in the responders than in the nonresponders after behavior therapy, and the baseline rCBF in the bilateral OFC correlated with changes in the responders’ clinical symptoms (Yamanishi et al., 2009). Nakao et al. (2005) found decreased BOLD responses in the DLPFC and bilateral OFC during symptom provocation after clinical improvement (Nakao et al., 2005). Yang et al. (2015) found that the higher regional homogeneity in the bilateral DLPFC and right OFC was decreased at resting‐state after 12 weeks of individual CBT (Yang et al., 2015). Morgieve et al. (2014) found that the hemodynamic response of the left OFC to obsession‐inducing images decreased after 3 months of CBT (Morgieve et al., 2014). Although these studies provide evidence of the effects of CBT on regional brain activity in the CSTC circuit, the question of whether CBT treatment can affect the intrinsic whole‐brain functional network remains unclear.

Spontaneous or intrinsic whole‐brain functional network properties can be analyzed using a graph‐theory approach with degree centrality (DC), which determines the number of voxels across the brain that strongly correlate with a target voxel, and describes the importance of the target voxel or hub in the whole‐brain network (Buckner et al., 2009; Zuo et al., 2012). The hubs in a brain network play an important role in efficient integration of communication over the whole‐brain, and have been used as an indicator of changes in resting‐state functional networks for OCD (Tian et al., 2016). Based on resting‐state functional magnetic resonance imaging (RS‐fMRI) and the DC approach, previous studies have found changes in the OCD‐related brain network hub not only in the CSTC circuit, but also distributed across the whole‐brain network, thereby indicating that whole‐brain functional network DC analysis is helpful in understanding the pathophysiology of OCD (Beucke et al., 2013; Hou et al., 2014; Tian et al., 2016).

Shin et al. (2014) explored the effects of treatment interventions on the whole‐brain functional network, and found that changes in DC in the right ventral frontal cortex correlated with improvement in obsessive–compulsive symptoms in OCD patients after selective serotonin reuptake inhibitor (SSRI) treatment (Shin et al., 2014). To the best of our knowledge, only one study has reported the predictive value of the intrinsic whole‐brain functional network for CBT effects using the DC approach in OCD in a resting‐state (Gottlich, Kramer, Kordon, Hohagen, & Zurowski, 2015). That study demonstrated that DC in the right basolateral amygdala correlated with the clinically positive effects of CBT, and suggested that DC in the right basolateral amygdala may be a candidate biomarker to predict the outcome of CBT. However, the study only used altered DC at baseline to predict OCD patients’ responses to CBT, and did not demonstrate changes in the intrinsic whole‐brain functional network properties in the resting‐state after successful CBT treatment. Building on this prior work, we aimed to explore the effects of CBT on the intrinsic whole‐brain functional network, based on resting‐state analysis of OCD patients.

We used the DC and functional connectivity approach with RS‐fMRI data to examine the effects of CBT treatment on the intrinsic whole‐brain functional network hub and connectivity in drug‐naive and noncomorbid OCD patients. Based on previous studies, we hypothesized that CBT would modulate the intrinsic whole‐brain network in brain hubs within the CSTC circuit. We also hypothesized that these changes may be associated with improvements in clinical presentation.

## 2 MATERIALS AND METHODS

### 2.1 Participants

We recruited 20 drug‐naive OCD outpatients from psychiatric outpatient clinics in Beijing Anding Hospital, and 20 age‐ and gender‐matched healthy controls (HCs) from the local community. All OCD patients and HCs were diagnosed by four experienced senior psychiatrists using the Structured Clinical Interview for DSM‐IV Axis I Disorders—Patient Edition and Nonpatient Edition, respectively.

All participants were assessed with the Yale‐Brown Obsessive–Compulsive Scale (Y‐BOCS) (Zhang, Meng, Cui, Gan, & Guo, 1996), 17‐item Hamilton Depression Rating Scale (HAMD‐17) (Tang & Zhang, 1984b) and Hamilton Anxiety Rating Scale (Tang & Zhang, 1984a). The inclusion criteria for OCD patients required a score of at least 16 on the Y‐BOCS; a score of less than 18 on the HAMD‐17; being right‐handed; and having no history of neurological illness or other major physical diseases; no history of Axis I psychiatric disorders other than OCD; no history of psychoactive substance use, alcohol dependence or alcohol abuse; and no previous or current use of psychotropic medication or psychotherapy. Ten OCD patients in the current study overlapped with the participants in the study our group performed previously (Yang et al., 2015). We used the Reliable Change Index (RCI) to define responders and nonresponders of CBT. The RCI is defined as an OCD patient's score change (baseline versus Week 12) in Y‐BOCS, divided by the standard error of the difference (Costa & Paula, 2015), and was calculated in the same manner as that undertaken by Kneebone, Andrew, Baker, and Knight (1998). The OCD patients were regarded as responders when the RCI was equal to or more than 1.96 (Kneebone et al., 1998). According to this criterion, 20 OCD patients were regarded as responders.

The HCs were screened using the Structured Clinical Interview for DSM‐IV Axis I Disorders—Nonpatient Edition. The inclusion criteria for HCs required no history of any neurological illness, major physical diseases or psychiatric disorders; no positive family history of major psychiatric disorders; and being right‐handed.

This study was approved by the Research Ethics Committee at Beijing Anding Hospital, Capital Medical University. Written informed consent was obtained from each participant.

### 2.2 CBT setting

The OCD patients undertook a 12‐week individual CBT program that consisted of 14 sessions (each session lasting 60 min). None of the OCD patients took psychoactive medications during the treatment period. Sessions 1 to 2 included information collecting, rapport building, evaluation, introduction to the CBT program and therapeutic setting, psycho‐education, normalization, and establishment of the therapeutic goal and plan. Sessions 3 to 6 included understanding patterns of automatic thoughts and behaviors’, a behavioral experiment, case conceptualization and cognitive reconstruction. Session 7 included introduction of exposure, creation of an anxiety hierarchy and planning of exposure. Sessions 8 to 12 included implementation of imaginative and field exposure, including therapist‐assisted and independent exposure exercises. Sessions 13 to 14 included review of the treatment process and consolidation of the treatment effect and relapse prevention (Yang et al., 2015).

### 2.3 Brain image data acquisition and image preprocessing

We obtained images with a Siemens Trio 3‐tesla scanner (Siemens Magnetom Trio; Erlangen, Germany) at the State Key Laboratory of Cognitive Neuroscience and Learning, Beijing Normal University, Beijing, China. Subjects lay supine, with foam pads and earplugs to reduce head motion and scanner noise. We defined resting‐state as the subject not engaging in any specific cognitive task during fMRI scanning (Biswal, Yetkin, Haughton, & Hyde, 1995). During RS‐fMRI acquisition, the subjects were instructed to be still, relax, close their eyes, avoid falling asleep and not think of anything in particular. We obtained the resting‐state functional scans using an echo‐planar imaging sequence with the following parameters: 33 axial slices, TR = 2,000 ms, TE = 30 ms, FA = 90°, thickness/gap = 3.5/0.6 mm, FOV = 200 × 200 mm, in‐plane resolution = 64 × 64, and 240 volumes in total (eight minutes). No participants displayed structural abnormalities during visual inspection.

We completed image preprocessing using Data Processing & Analysis for (Resting‐State) Brain Imaging (DPABI) (Yan, Wang, Zuo, & Zang, 2016) and BrainNet Viewer software (Xia, Wang, & Yong, 2013). The preprocessing procedures included removing the first 10 time‐points, slice timing, head motion correction, nuisance covariates regression (including the white matter signal, cerebrospinal fluid signal and Friston 24 parameter model), filtering with a band‐pass filter (0.01–0.1 Hz) and normalization to the Montreal Neurological Institute space using standard echo‐planar template images. We conducted the scrubbing procedure, and excluded any volume with a frame‐wise displacement value exceeding 0.5, together with one preceding and two subsequent volumes (Power, Barnes, Snyder, Schlaggar, & Petersen, 2012). There were no group × time effects on the mean frame‐wise displacement (F = 0.225, *p* = .637). Post hoc analysis showed that there was no difference in frame‐wise displacement between the OCD patients and HCs at baseline, and between the OCD patients before and after CBT treatment (Table 1).

**Table 1:** Demographic and clinical data of obsessive compulsive disorder and healthy controls

	OCD (*n *= 20) Baseline	HCs (*n *= 20) Baseline	OCD (*n *= 20) Week 12	HCs (*n *= 20) Week 12	*p*
Age (years)	30.35 ± 7.49	30.55 ± 7.84	30.65 ± 7.42	30.80 ± 7.87	.935^a^
Gender (male/female)	13/7	13/7	13/7	13/7	1.000^a^
Education level (years)	15.15 ± 3.36	15.75 ± 2.92	15.15 ± 3.36	15.75 ± 2.92	.550^a^
Illness duration (months)	97.65 ± 99.98		100.56 ± 99.98		
Y‐BOCS score					
Total	23.90 ± 5.62	0.75 ± 0.97	11.95 ± 6.95	0.75 ± 0.97	.000^b^/1.000^c^
Obsessions	11.85 ± 5.20	0.40 ± 0.60	5.30 ± 4.23	0.40 ± 0.60	.000^b^/1.000^c^
Compulsions	12.05 ± 3.33	0.30 ± 0.47	6.65 ± 3.67	0.35 ± 0.49	.000^b^/.330^c^
HAMD score	8.40 ± 4.38	1.60 ± 1.27	3.70 ± 3.42	1.60 ± 1.23	.000^b^/1.000^c^
HAMA score	11.55 ± 5.66	1.95 ± 1.10	4.55 ± 4.17	2.05 ± 1.23	.000^b^/.761^c^
FD	0.07 ± 0.03	0.08 ± 0.05	0.07 ± 0.03	0.09 ± 0.06	.328^a^/.769^b^

OCD, obsessive‐compulsive disorder; HCs, healthy controls; Y‐BOCS, Yale‐Brown Obsessive–Compulsive Scale; HAMD, 17‐item Hamilton Depression Rating Scale; HAMA, Hamilton Anxiety Rating Scale; FD, frame‐wise displacement.

Data are presented as mean ± standard deviation or number. The variables, gender, and handedness were analyzed using chi‐square test, while other variables were analyzed using independent‐ sample *t*‐test.

a Indicate the *p* values for the comparisons between the OCD patients and the healthy controls at baseline.

b Indicate the *p* values for the comparisons between the OCD patients at baseline and the patients after 12 weeks of treatment.

c Indicate the *p* values for the comparisons between the healthy controls at baseline and the healthy controls after 12 weeks.

### 2.4 Network degree centrality analysis

Every voxel in the brain is a node, while the functional connectivity of any two voxels is an edge. For each voxel in the brain, we calculated its functional connectivity with all other brain voxels, and summed all these correlations as the voxel's DC. We determined the DC maps in the entire brain through the correlation of each voxel's time series to all other voxels’ time series to construct a whole‐brain connectivity matrix for each participant within the grey matter templates of the DPABI software, and by counting the number of voxels where the correlation with the time series exceeded a predefined statistical threshold (DC maps were computed using 0.3 as the correlation threshold) (Beucke et al., 2013; Zuo et al., 2012). We further smoothed these maps with a 6‐mm full‐width at half‐maximum Gaussian kernel, and normalized to standard z‐scores. We used one‐sample *t*‐tests to produce the within‐group whole‐brain DC in the HC and OCD groups at baseline and 12 weeks, respectively. We performed two‐way analysis of covariance (ANCOVA) and post hoc analyses to determine group × time interactions, and the main effects of group (OCD and HC groups) and time (Weeks 12 and 0) using DC maps in a whole‐brain voxel‐wise approach. We used SPM8 software to implement the ANCOVA analysis, with full factorial design (factors: group and time; levels: baseline and week 12). We restricted the ANCOVA within a grey mask that excluded the voxels showing significant DC map changes in the HC group over time. We made the mask by performing a paired *t*‐test on the DC map of the HC group between 12 weeks and baseline, with a threshold of *p *<* *.05 (Gaussian random field [GRF] correction). As an approach for multiple comparison corrections, GRF correction has been used for exploratory network analysis in previous research (Wu et al., 2015; Yuan et al., 2016). The result of the ANCOVA for DC was threshold at a voxel *p*‐value <.001 and a cluster *p*‐value <.05 (GRF corrected, two‐tailed). We recomputed the DC maps with two different correlation thresholds (0.2 and 0.4) and preformed the respective statistical analyses to test the reproducibility of the results.

### 2.5 Functional connectivity

To examine in detail the resting‐state functional connectivity (RSFC) alterations in OCD patients, we performed seed‐based functional connectivity analyses with left DLPFC as seed according to the result of network degree centrality analysis. To avoid circular analysis of the same dataset and double‐dipping, seed regions need to be defined independently from DC analysis (Kriegeskorte, Simmons, Bellgowan, & Baker, 2009). For this reason, the left DLPFC mask was made as seed using a mask of the left middle frontal gyrus restricted to Brodmann areas 9, 10, and 46 with Wake Forest University PickAtlas (http://fmri. wfubmc.edu/software/PickAtlas), which was used in previous research (Hutcherson, Plassmann, Gross, & Rangel, 2012) (Figure3). We obtained the reference time course by averaging the time series of all voxels in the left DLPFC mask, and then conducted Pearson's correlation analyses between the seed (left DLPFC) reference time courses and the time series of all other brain voxels in a voxel‐wise manner, with the six head motion parameters, global mean time courses, white matter time courses and cerebrospinal fluid time courses as nuisance factors. We used one‐sample *t*‐tests to assess within‐group differences in the RSFC maps in the HC and OCD groups at baseline and 12 weeks, respectively. We performed ANCOVA and post hoc analyses on the RSFC maps for each seed. We conducted the analyses within a mask that excluded voxels that showed significant time differences in RSFC in the HC group. We made this mask by performing a paired *t*‐test on the RSFC of the HC group between 12 weeks and baseline, with a threshold of *p *<* *.05 (GRF correction). We defined the significance level for RSFC results as a voxel *p*‐value <.001 and a cluster *p*‐value <.05 (GRF corrected, two‐tailed).

### 2.6 Statistical analysis

In addition to the statistical analyses described above, we performed independent *t*‐tests and chi‐square tests on the demographic and clinical data using SPSS Version 13.0 (SPSS Inc., Chicago, IL, USA). We performed correlation analyses between the percentage reduction changes in the Y‐BOCS score and changes in the DC and seed‐based RSFC at the voxel‐wise level for the entire brain. We used a threshold of *p *<* *.05/3 (.017) (Bonferroni corrected) in this context, controlling for three correlations (Y‐BOCS total score, obsessions score and compulsions score).

## 3 RESULTS

### 3.1 Sample characteristics

Table 1 displays the demographic and clinical data. There were no significant differences in age, gender, handedness, education or frame‐wise displacement between the OCD and HC groups (all *p *>* *.05). OCD‐related symptoms decreased significantly after CBT treatment in all OCD patients (all *p *<* *.0001).

### 3.2 Network degree centrality

**Figure 1:**
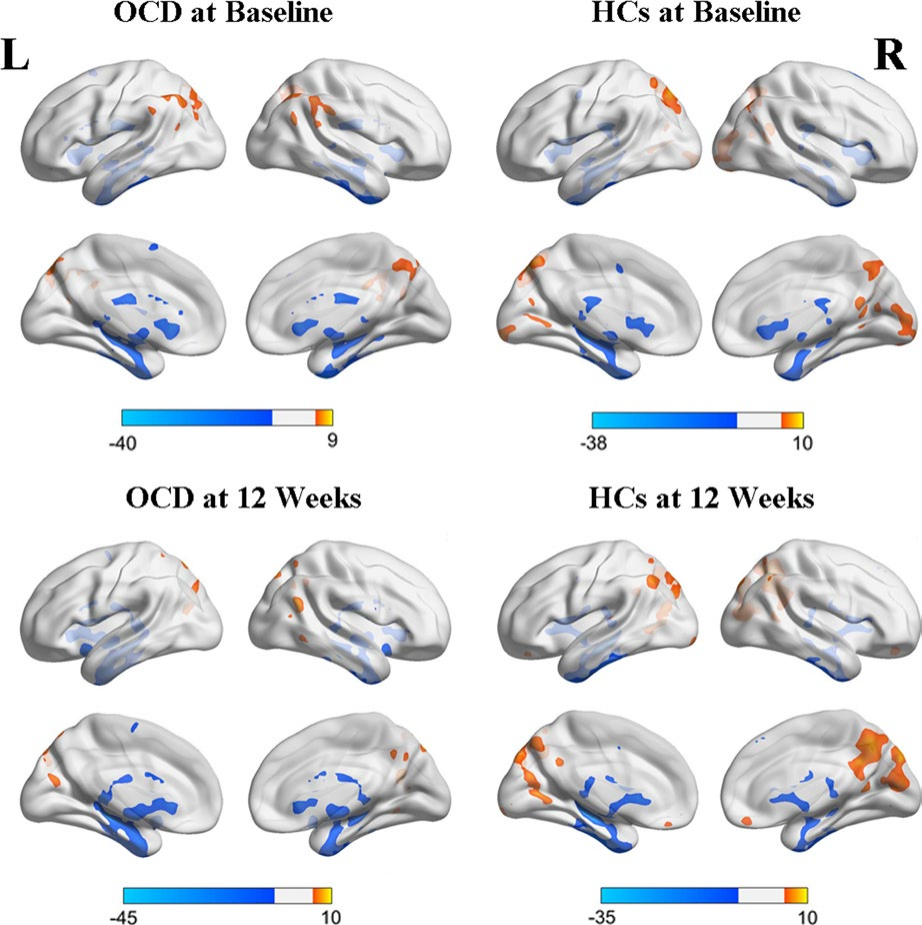
Within‐group whole‐brain degree centrality in OCD, HCs at baseline and 12 weeks, respectively. The threshold was a voxel *p*‐value <.001, a cluster *p*‐value <.05, two‐tailed (GRF correction). L, left side; R, right side

**Figure 2:**
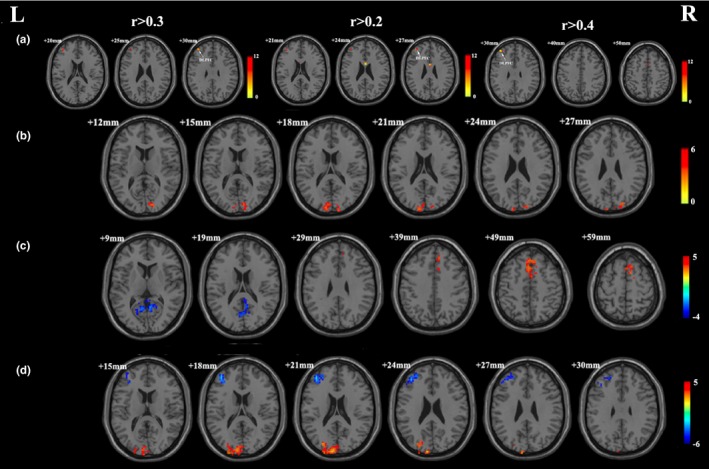
(a) Significant group × time interactions on degree centrality with 0.3, 0.2, and 0.4 as correlation threshold respectively. (b) Significant effect of time on degree centrality. (c) Brain regions showing significant difference in degree centrality between OCD patients and the healthy controls at baseline. (d) Brain regions showing significant difference in degree centrality in OCD patients following CBT. L, left side; R, right side. The threshold was a voxel *p*‐value <.001, a cluster *p*‐value <.05, two‐tailed (GRF correction) for a and b; and voxel *p*‐value <.05, a cluster *p*‐value <.05, two‐tailed (GRF correction) for c and d

We calculated DC for the 20 CBT responders and 20 HCs. The DC patterns were similar across the OCD and HC groups (Table 2, Figure 1). We observed significant group × time interactions on DC in the left DLPFC (Table 3, Figure 2a), and found a significant effect of time on DC in the bilateral superior occipital gyrus (Table 3, Figure 2b). These results were largely preserved after accounting for the effect of correlation thresholds (Figure 2a). Voxel‐wise post hoc analysis at whole‐brain showed that the OCD patients had decreased DC in the right lingual gyrus, and increased DC in the right supplementary motor area, compared with the HCs group at baseline (Table 4, Figure 2c); as well as decreased DC in the left DLPFC and increased DC in the left superior occipital gyrus following CBT treatment (Table 5, Figure 2d).

**Table 2:** Brain regions showing significant within‐group effects of DC in the present samples

Brain regions	Brodmann area	Cluster size (voxels)	MNI coordinates (x, y, z)	Peak *t* value
OCD patients at baseline				
OFC	11	65	27, 60, 0	6.565
Middle frontal gyrus		138	48, 39, 30	7.864
Precentral cortex	6	292	−51, 3, 42	7.526
White matter		14,893	42, −30, −12	−39.870
OCD patients at week 12				
Superior medial frontal gyrus		112	0, 63, 6	6.118
Middle frontal gyrus	6	91	51, −9, 54	6.783
Superior temporal gyrus	48	128	66, 3, 0	10.633
Superior temporal gyrus	22	217	−66, −9, 3	9.031
Middle cingulate cortex		88	0, 12, 39	8.103
White matter		15,170	−21, −45, −39	−45.563
HCs at baseline				
OFC	11	338	0, 54, −9	6.773
Superior temporal gyrus	38	259	54, 21, −12	7.695
Superior temporal gyrus	38	268	−54, 18, −18	7.752
Supplementarymotorarea	6	176	6, −12, 78	8.829
White matter		17,251	−30, −42, 3	−39.944
HCs at week 12				
Superior temporal gyrus	22	103	−60, −27, 12	9.206
Superior temporal gyrus	38	179	60, 12, −6	9.388
Superior temporal gyrus	38	99	−48, 21, −12	9.243
Inferiortemporalgyrus	20	16,552	−42, −30, −15	−33.652
Rectus	11	390	0, 48, −21	7.143
Paracentral_Lobule	4	103	−6, −30, 78	6.908

MNI, Montreal Neurological Institute; OCD, obsessive‐compulsive disorder; OFC, orbitofrontal cortex.

The threshold was set at a voxel *p* value <.001, and a cluster *p* value <.05 (GRF corrected, two‐tailed).

**Table 3:** The results of ANCOVA on degree centrality

Brain regions	Brodmann area	Cluster size (voxels)	MNI coordinates (x, y, z)	*F*_(df)_/*t*	*p*
Brain regions showing significant group × time interaction on degree centrality					
DLPFC	46	41	−39, 48, 30	11.583_(1,39)_	<.05
Brain regions showing significant effect of time on degree centrality					
Superior occipital gyrus	19	63	24, −84, 27	5.023	<.05
Superior occipital gyrus	18	44	−12, −93, 18	4.968	<.05

MNI, Montreal Neurological Institute; DLPFC, dorsolateral prefrontal cortex.

The threshold was set at a voxel *p* value <.001, and a cluster *p* value <.05 (GRF corrected, two‐tailed).

**Table 4:** Brain regions showing significant difference in degree centrality and RSFC between OCD patients and the healthy controls at baseline

Brain regions	Brodmann area	Cluster size (voxels)	MNI coordinates (x, y, z)	Peak *t* value
Brain regions showing significant difference in degree centrality				
Lingual gyrus	30	267	6, −54, 9	−3.870
Supplementary motor area	6	307	9, 9, 63	4.201
Brain regions showing significant difference in RSFC				
OFC	11	198	14, 48, −8	3.964

MNI, Montreal Neurological Institute; RSFC, resting‐state functional connectivity; OFC, orbitofrontal cortex.

The threshold was set at a voxel *p* value <.05, and a cluster *p* value <.05 (GRF corrected, two‐tailed).

**Table 5:** Brain regions showing significant difference in degree centrality in OCD patients following CBT

Brain regions	Brodmann area	Cluster size (voxels)	MNI coordinates (x, y, z)	Peak *t* value
DLPFC	46	127	−39, 45, 25	−5.806
Superior occipital gyrus	18	143	−9, −96, 21	5.034

MNI, Montreal Neurological Institute; DLPFC, dorsolateral prefrontal cortex.

The threshold was set at a voxel *p* value <.05, and a cluster *p* value <.05 (GRF corrected, two‐tailed).

### 3.3 Seed‐based RSFC

**Figure 3:**
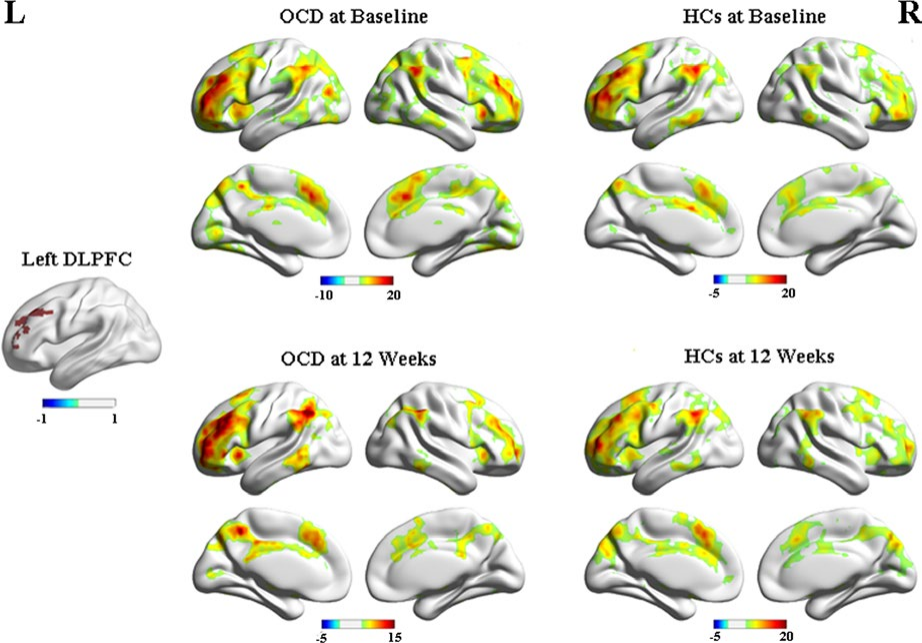
Within‐group resting‐state functional connectivity with left DLPFC in OCD, HCs at baseline and 12 weeks, respectively. The threshold was a voxel *p*‐value <.001, a cluster *p*‐value <.05, two‐tailed (GRF correction). L, left side; R, right side

**Figure 4:**
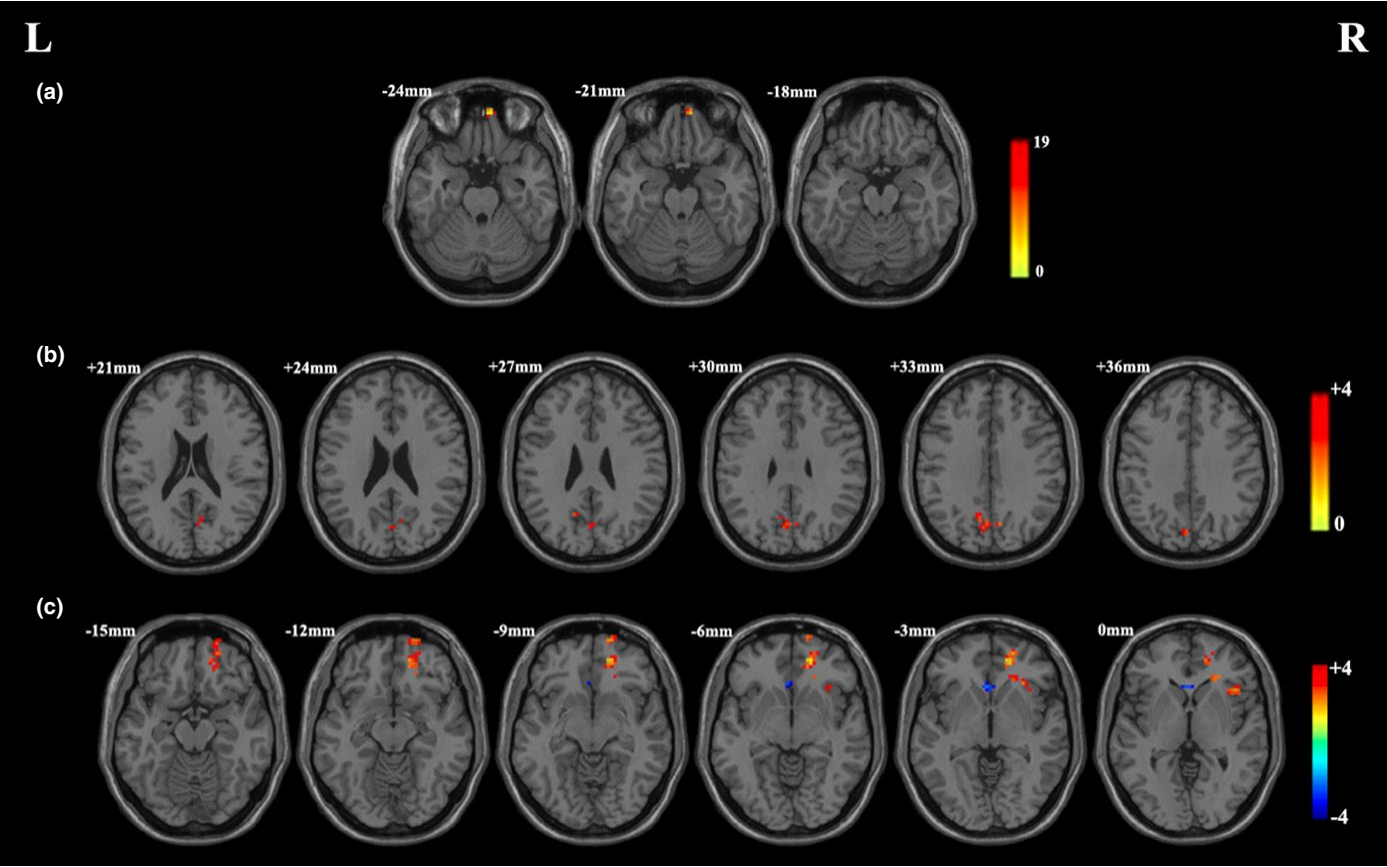
(a) Significant group × time interactions on resting‐state functional connectivity. (b) Significant effect of time on resting‐state functional connectivity. (c) Brain regions showing significant difference in resting‐state functional connectivity between OCD patients and the healthy controls at baseline. L, left side; R, right side. The threshold was a voxel *p*‐value <.001, a cluster *p*‐value <.05, two‐tailed (GRF correction) Figure 2a,b; and voxel *p*‐value <.05, a cluster *p*‐value <.05, two‐tailed (GRF correction) for Figure 2c

The RSFC patterns were similar across the OCD and HCs groups (Figure 3). The region that showed significant group × time interactions on the RSFC of the left DLPFC was located in the right OFC (Table 6, Figure 4a), and we found a significant effect of time on RSFC in the right precuneus (Table 6, Figure 4b). Voxel‐wise post hoc analysis at whole‐brain revealed that the OCD patients had increased RSFC between the left DLPFC and right OFC, compared with the HCs group at baseline (Table 4, Figure 4c), while no brain regions showed different RSFC in the OCD patients following CBT treatment.

**Table 6:** The results of ANCOVA on resting‐state functional connectivity

Brain regions	Brodmann area	Cluster size (voxels)	MNI coordinates (x, y, z)	*F*_(df)_/*t*	*p*
Brain regions showing significant group×time interaction on RSFC					
OFC	11	10	9, 56, −24	19.942_(1,39)_	<.05
Brain regions showing significant effect of time on RSFC					
Precuneus		38	9, −69, 33	3.818	<.05

MNI, Montreal Neurological Institute; RSFC, resting‐state functional connectivity; OFC, orbitofrontal cortex.

The threshold was set at a voxel *p* value <.001, and a cluster *p* value <.05 (GRF corrected, two‐tailed).

### 3.4 Association between changes in DC and RSFC and the clinical symptom changes of OCD

**Figure 5:**
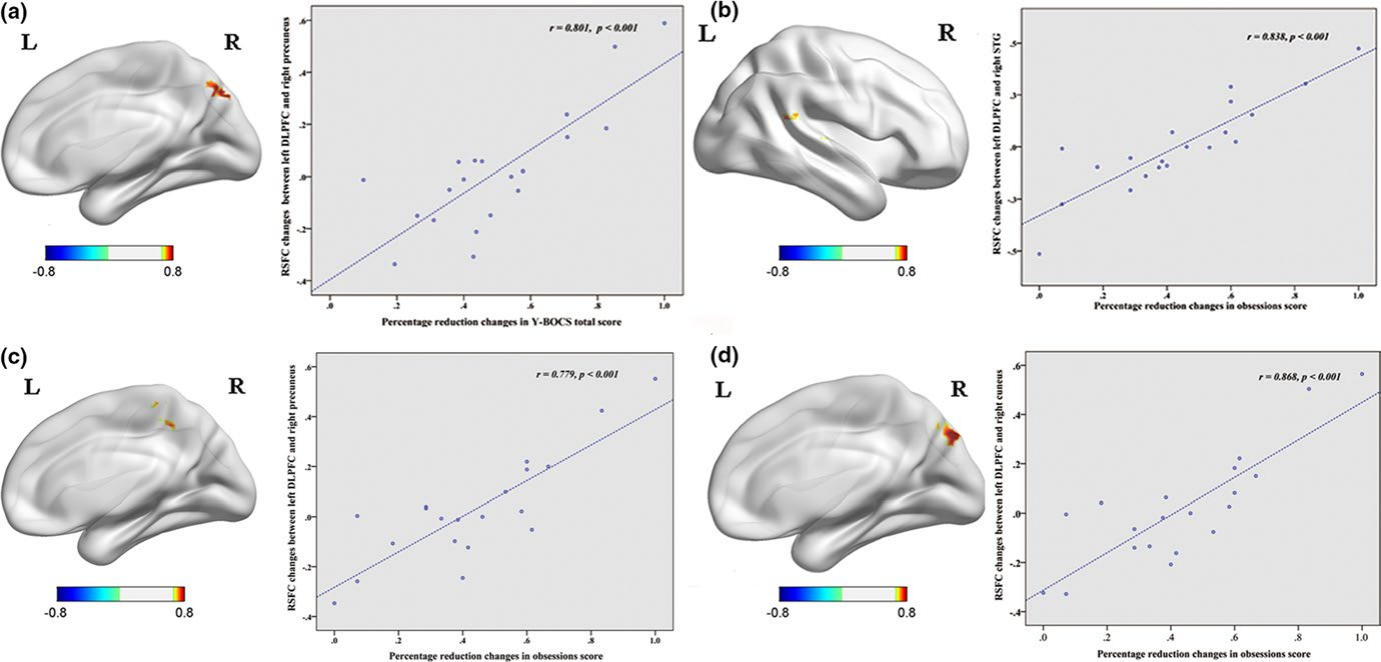
(a) Positive correlation between RSFC changes (left DLPFC and right precuneus) and percentage reduction changes in Y‐BOCS total score. (b) Positive correlation between RSFC changes (left DLPFC and right superior temporal gyrus) and percentage reduction changes in obsessions score. (c) Positive correlation between RSFC changes (left DLPFC and right precuneus) and percentage reduction changes in obsessions score. (d) Positive correlation between RSFC changes (left DLPFC and right cuneus) and percentage reduction changes in obsessions score. L, left side; R, right side. The threshold was a voxel *p*‐value <.001, a cluster *p*‐value <.05, two‐tailed (GRF correction)

DC changes at whole‐brain exhibited no correlation with improvements in clinical symptoms. RSFC changes between the left DLPFC and right precuneus positively correlated with percentage reduction changes in the Y‐BOCS total score (*r* = .801; *p *<* *.001) (Table 7, Figure 5a); RSFC changes between the left DLPFC and right superior temporal gyrus (Figure 5b), precuneus (Figure 5c) and cuneus (Figure 5d) positively correlated with percentage reduction changes in the Y‐BOCS obsessions score (*r* = .838, *r* = .779, *r* = .868; *p *<* *.001) (Table 7).

**Table 7:** Correlation between RSFC changes and OCD clinical symptom changes

Brain regions	Brodmann area	Cluster size (voxels)	MNI coordinates (x, y, z)	*r*
Precuneus[Link]	7	77	6, −75, 39	.801
Superior temporal gyrus[Link]	42	63	57, −42, 18	.838
Precuneus[Link]		66	9, −45, 48	.779
Cuneus[Link]	19	104	15, −81, 39	.868

MNI, Montreal Neurological Institute.

Correlation between RSFC changes of the whole brain and percentage reduction changes in Y‐BOCS total score,^a^ and obsessions score^b^ in OCD patients. The threshold was set at a voxel *p* value <.001, and a cluster *p* value <.05 (GRF corrected, two‐tailed).

## 4 DISCUSSION

CBT treatment has been proven effective in many randomized controlled trial studies (Olatunji et al., 2014) and, in this study, the OCD patients did not receive any other treatment, such as psychoactive medication. Thus, the CBT was most likely to have caused the improvement in the OCD patients’ clinical symptoms. The changes in the resting‐state intrinsic whole‐brain functional network properties found in this study were also due to the CBT treatment.

To the best of our knowledge, this is the first study to investigate the effect of CBT treatment on intrinsic whole‐brain functional network hubs and connectivity in drug‐naive and noncomorbid OCD patients in a resting‐state. Consistent with our hypothesis, the treatment‐related changes were observed in the brain hub of the DLPFC, which is involved in the CSTC circuit of OCD patients. We specifically found that 14 sessions (12 weeks) of CBT treatment decreased the DC in the left DLPFC and normalized the higher RSFC between the left DLPFC and the right OFC in OCD patients.

The prefrontal cortex is at the highest level in the cognitive control hierarchy (Goldman‐Rakic, 1995), and the DLPFC is considered important for cognitive flexibility and executive planning, which are impaired in OCD patients (Menzies et al., 2008). Increased regional homogeneity and decreased gray matter volume in the left DLPFC were observed in OCD patients in our previous research with a different sample (Chen et al., 2016), as well as in meta‐analytic studies (Peng et al., 2012; Rotge et al., 2010). Although we did not find abnormal DC in the left DLPFC in the OCD patients at baseline, increased cerebral blood flow and glucose metabolism in the DLPFC were reported among OCD patients in previous studies (Nakao et al., 2009; Remijnse et al., 2009; Swedo et al., 1989), thereby suggesting the important role of the DLPFC in the pathophysiological mechanism of OCD.

CBT treatment helps OCD patients develop more effective cognitive strategies to adapt to their external and changing environment (Moras, 2006). Previous studies have found that the higher rCBF and regional homogeneity in the left DLPFC decreased after CBT treatment (Yamanishi et al., 2009; Yang et al., 2015), and the abnormalities of grey matter volume in DLPFC negatively affected the CBT outcomes (Tsuchiyagaito et al., 2017). The current study's finding that successful CBT treatment decreased the DC in the left DLPFC indicates that the numbers of voxels across the brain that strongly correlate with the left DLPFC decreased, which may be related to the much lower effort required to control intrusive thoughts and repetitive behavior in OCD patients after CBT. CBT treatment can cause synaptic changes through restructuring thoughts and modifying feelings and behavior (Moras, 2006). The left DLPFC may be a connective hub of synaptic change, and changes in connectivity via this hub could be a mechanism involved in the therapeutic effects of CBT.

At the same time, we found increased RSFC between the left DLPFC and right OFC in the OCD patients at baseline, and normalized after CBT treatment. The OFC plays a key role in the pathophysiology of OCD and is involved in response inhibition and behavior suppression (Menzies et al., 2008). Increased activity at resting‐state and decreased activity at task‐state in the OFC have been observed in OCD patients (Hou et al., 2014; Ping et al., 2013). DLPFC and OFC may work together to inhibit intrusive thoughts and repetitive behavior; thus, increased RSFC between these two brain regions may lead to a dysfunctional strategy to copy with the clinical symptoms of OCD. Previous studies have emphasized the important role of OFC in CBT treatment for OCD patients (Morgieve et al., 2014; Nakao et al., 2005; Yamanishi et al., 2009; Yang et al., 2015). In the current study, CBT may have alleviated cognition and compulsive behavior by acting on the cortical regions (DLPFC and OFC), thereby resulting in amelioration of the obsession and compulsion (Nakao, Okada, & Kanba, 2014). The results of the current study indicate that the modulation of DLPFC connectivity by CBT treatment could synchronously lead to alterations in other brain regions (such as the OFC), which may play an important role in the CSTC circuit pathology underlying OCD.

Inconsistent with our hypothesis, the ANCOVA results in this study showed that a significant effect of time on DC was found in the bilateral superior occipital gyrus. Voxel‐wise post hoc analysis at whole‐brain revealed that the DC in the occipital gyrus was decreased at baseline (right lingual gyrus) and increased after CBT treatment (left superior occipital gyrus) in the OCD patients. In addition to mediating visual word processing, the occipital gyrus is involved in processing emotionally charged visual stimuli (Szeszko et al., 2005). Decreased regional homogeneity, fractional amplitude of low‐frequency fluctuation and functional connectivity strength in the occipital gyrus have been reported in OCD patients at resting‐state (Hou et al., 2014; Ping et al., 2013; Qiu et al., 2017; Tian et al., 2016). The present results combined with previous studies suggest that the occipital gyrus may be involved in the pathophysiology of OCD. CBT increased the numbers of voxels strongly correlated with the occipital gyrus, which may be related to the improved emotional processing ability among OCD patients caused by CBT. Based on the results of this study, we speculate that CBT may also affect the brain regions outside the CSTC circuit, such as the occipital gyrus. However, interpretation of the current abnormal results of voxel‐wise post hoc analysis at whole‐brain should be undertaken cautiously because these results were not upheld after strict multiple comparison correction (a voxel *p‐*value <.001 and a cluster *p*‐value <.05, GRF corrected).

In addition, we found a positive correlation between clinical improvement in OCD and RSFC changes between the left DLPFC and right precuneus, cuneus and superior temporal gyrus, which is involved in the default mode network (DMN) (Greicius, Krasnow, Reiss, & Menon, 2003). The DLPFC is an important brain region for the cognitive control network or fronto‐parietal network (Liao et al., 2010); thus, our results suggest that the RSFC changes between the cognitive control network and DMN correlated with clinical improvement in OCD. The DMN could not “switch off” when attention was needed to direct to external stimuli in a cognitive task, which may lead to difficulties in separating from internally generated intrusive thoughts, and subsequently cause thought–action fusion. CBT may improve the reduced negative relationship between the cognitive control network and DMN in OCD patients revealed by previous research (Stern et al., 2012) and, to support competitive relationships between these two networks, help OCD patients separate from internally‐focused intrusive thoughts to external attention processes (Stern et al., 2012). Further research is needed to explore the changes in relationship between the cognitive control network and DMN before and after CBT treatment to clarify the effect of CBT on the relationship between these two networks.

A previous study reported that DC changes in the right ventral frontal cortex correlated with clinical improvements in OCD patients after SSRI treatment (Shin et al., 2014), which suggests that SSRIs may affect the intrinsic network connectivity associated with affective circuits. Additionally, our results suggest that CBT treatment may alter the intrinsic network connectivity associated with cognitive circuits, involving brain regions such as the DLPFC and OFC. Taken together, the above findings indicate that SSRIs and CBT may exert their respective therapeutic effects in OCD patients through mechanisms that differentially influence intrinsic neural circuits.

Previous studies have emphasized the important role of other brain regions in the CSTC circuit (dACC, caudate and thalamus) in CBT treatment for OCD patients. CBT helps OCD patients develop more effective cognitive tactics to reappraise and suppress negative emotions, which may be related with increased dACC activity after intensive CBT (Saxena et al., 2009). The OCD symptom improvement completed through CBT may be accompanied by functional changes of the caudate nucleus (Schwartz et al., 1996). The thalamus is known to be a possible mediator of CBT effects (Atmaca et al., 2016), whereby the decreased thalamic activity may be a final common pathway for improvement in OCD (Saxena et al., 2009). However, the present study failed to identify the role of these brain regions in CBT among OCD patients. The reproducibility of neuroimaging findings may decrease because of the intrinsically low statistical power of the relatively small sample size (Button et al., 2013). Moreover, our research did not find the amygdala connectivity in CBT treatment observed by Gottlich et al. (2015). This question of dACC, caudate, thalamus and amygdala functioning should be explored further in future studies, using a uniform CBT setting with larger sample sizes.

Several limitations of the current study should be acknowledged. First, the sample size of OCD patients in this study was relatively small because it is difficult for patients to receive only CBT, without medications. We are continuing to undertake this study to accumulate more samples and explore the neural mechanism of CBT. Second, the structural changes (grey matter and white matter) underlying the treatment effects of CBT need to be explored using multimodal imaging data analysis. Finally, although between‐group comparisons revealed that changes in the whole‐brain functional network may underlie improvements in OCD symptom severity, this finding has limited clinical application because clinicians need to predict the effect of CBT at the individual level, rather than the group level. In future studies, we will attempt to enhance clinical utility by applying novel analytical methods (machine learning techniques) to predict the effects of CBT on a given patient, with the goal of developing personalized treatment solutions.

In summary, this study demonstrated that successful CBT treatment can modulate intrinsic functional network hub changes in the CSTC circuit in OCD patients. Cognitive control network and DMN connectivity may be a potential imaging biomarker for evaluating CBT treatment for OCD. This study offers new insights into the effects of CBT at the neural circuitry level.

## CONFLICT OF INTEREST

The authors have no conflict of interest to declare.
